# Supplementary material for: Identification of novel HPFH-like mutations by CRISPR base editing that elevate the expression of fetal hemoglobin
Source: eLife. 2022 Feb 11;11:e65421. doi: 10.7554/eLife.65421 (PMC8865852; doi:10.7554/eLife.65421)
Supplement: Figure 5—source data 1. — Lanes 1, 4, 7, and 10 contain nuclear extracts from COS cells transfected with a pcDNA3 empty vector. Lanes 2–3, 5–6, 8–9, and 11–12 contain nuclear extracts from COS cells overexpressing KLF1. Binding of KLF1 to the –123T > C/–124T > C hereditary persistence of fetal hemoglobin (HPFH) mutant probe can be observed in lane 11, with a super shift of KLF1 in the presence of anti-KLF1 antibody in lane 12. [file elife-65421-fig5-data1.zip › Figure 5-source data1/Figure 5-source data1(labelled).pdf]

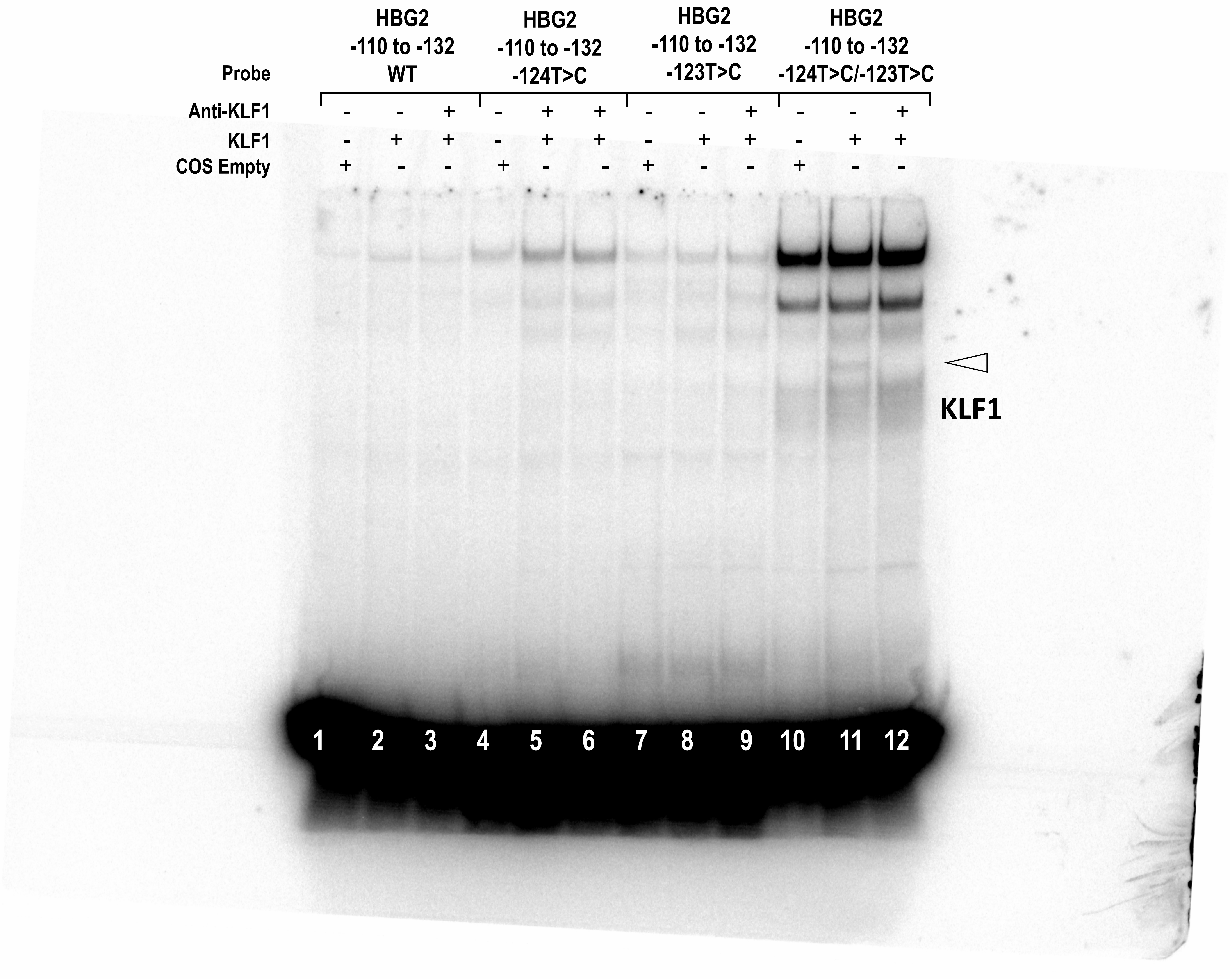

EMSA showing KLF1 binding to -123T>C/-124T>C probe but failing to bind to -124 T>C probe, -123T>C probe and WT probe with the -123 T/-124 T region of the HBG promoter in vitro. Lanes 1, 4 ,7 and 10 contain nuclear extracts from COS cells transfected with a pcDNA3 empty vector. Lanes 2-3, 5-6, 8-9 and 11-12 contain nuclear extracts from COS cells overexpressing KLF1. Binding of KLF1 to the -123T>C/-124T>C HPFH mutant probe can be observed in lane 11, with a super shift of KLF1 in the presence of anti-KLF1 antibody in lane 12.
