# Supplementary figures and images for: Identification of novel HPFH-like mutations by CRISPR base editing that elevate the expression of fetal hemoglobin
Source: eLife. 2022 Feb 11;11:e65421. doi: 10.7554/eLife.65421 (PMC8865852; doi:10.7554/eLife.65421)

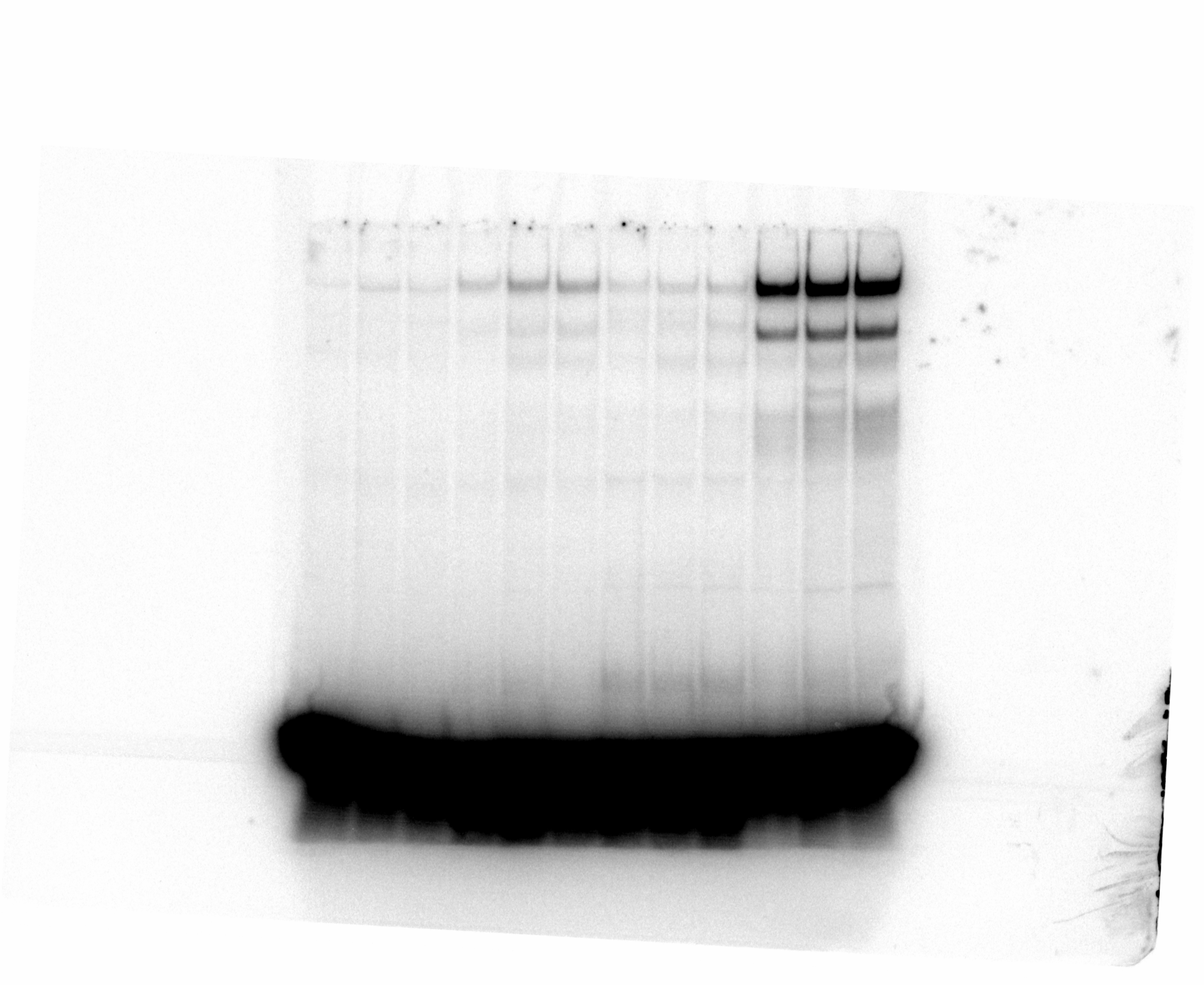

Supplement: Figure 5—source data 1. — Lanes 1, 4, 7, and 10 contain nuclear extracts from COS cells transfected with a pcDNA3 empty vector. Lanes 2–3, 5–6, 8–9, and 11–12 contain nuclear extracts from COS cells overexpressing KLF1. Binding of KLF1 to the –123T > C/–124T > C hereditary persistence of fetal hemoglobin (HPFH) mutant probe can be observed in lane 11, with a super shift of KLF1 in the presence of anti-KLF1 antibody in lane 12. [file elife-65421-fig5-data1.zip › Figure 5-source data1/Figure 5-source data1.jpg]

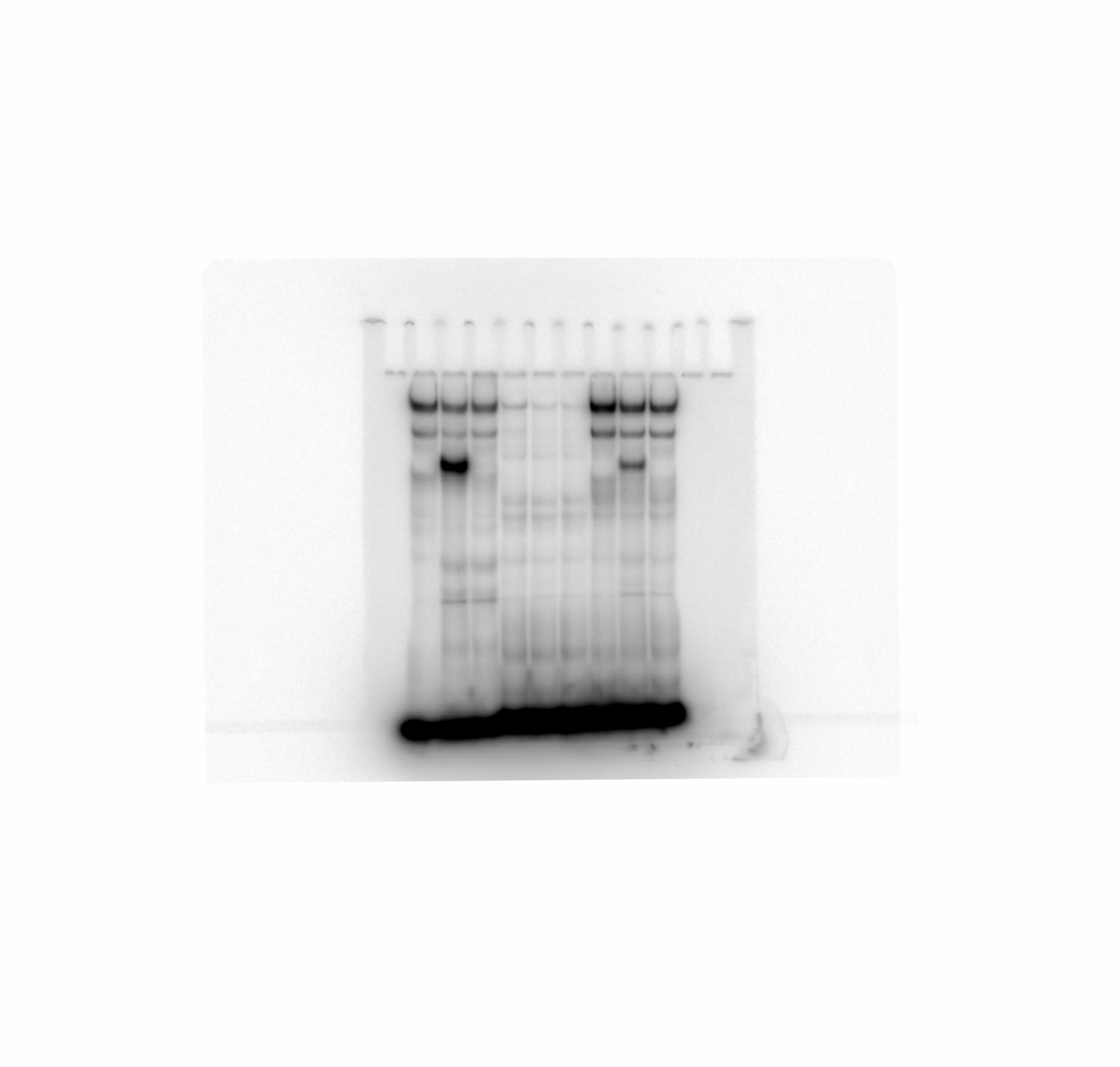

Supplement: Figure 5—figure supplement 1—source data 1. — Lanes 1–3 contain the Hbbt1-CACCC as positive control, lanes 4–6 contain the WT probe for the −123,–124 site (−132 to –110 bp) and lanes 7–9 contain the hereditary persistence of fetal hemoglobin (HPFH) −123/–124T > C mutant probe. Lanes 1, 4, and 7 contain nuclear extracts from COS cells transfected with a pcDNA3 empty vector. Lanes 2–3, 5–6, and 8–9 contain nuclear extracts from COS cells overexpressing KLF1. Binding of KLF1 to the −123/–124T > C HPFH mutant probe can be observed in lane 8, with a super shift of KLF1 with an anti-KLF1 antibody in lane 9. [file elife-65421-fig5-figsupp1-data1.zip › Figure 5-figure supplement 1-source data1/Figure 5-figure supplement 1-source data1.jpg]
