# Supplementary material for: Identification of novel HPFH-like mutations by CRISPR base editing that elevate the expression of fetal hemoglobin
Source: eLife. 2022 Feb 11;11:e65421. doi: 10.7554/eLife.65421 (PMC8865852; doi:10.7554/eLife.65421)
Supplement: Figure 5—figure supplement 1—source data 1. — Lanes 1–3 contain the Hbbt1-CACCC as positive control, lanes 4–6 contain the WT probe for the −123,–124 site (−132 to –110 bp) and lanes 7–9 contain the hereditary persistence of fetal hemoglobin (HPFH) −123/–124T > C mutant probe. Lanes 1, 4, and 7 contain nuclear extracts from COS cells transfected with a pcDNA3 empty vector. Lanes 2–3, 5–6, and 8–9 contain nuclear extracts from COS cells overexpressing KLF1. Binding of KLF1 to the −123/–124T > C HPFH mutant probe can be observed in lane 8, with a super shift of KLF1 with an anti-KLF1 antibody in lane 9. [file elife-65421-fig5-figsupp1-data1.zip › Figure 5-figure supplement 1-source data1/Figure 5-figure supplement 1-source data1 (labelled).pdf]

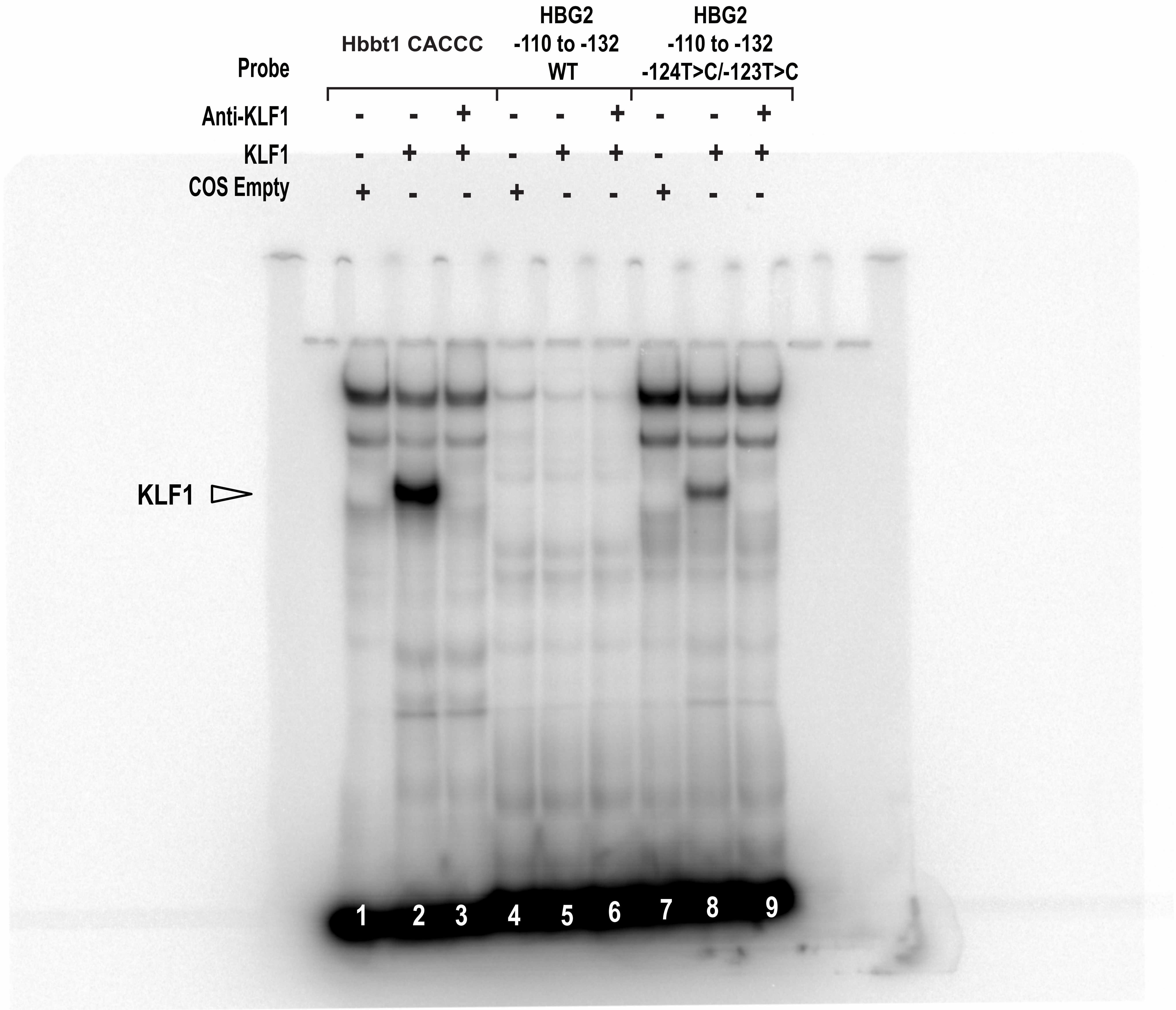

EMSA showing the binding of KLF1 to the -123T>C/-124T>C probe but fails to bind to a WT probe containing the -123/-124 region of the HBG promoter in-vitro. Lanes 1-3 contain the Hbbt1-CACCC as positive control, lanes 4-6 contain the WT probe for the -123, -124 site (-132 to -110 bp) and lanes 7-9 contain the HPFH -123/-124T>C mutant probe. Lanes 1, 4 and 7 contain nuclear extracts from COS cells transfected with a pcDNA3 empty vector. Lanes 2-3, 5-6 and 8-9 contain nuclear extracts from COS cells overexpressing KLF1. Binding of KLF1 to the -123/-124T>C HPFH mutant probe can be observed in lane 8, with a super shift of KLF1 with an anti-KLF1 antibody in lane 9.
