# Supplementary material for: Identification of novel HPFH-like mutations by CRISPR base editing that elevate the expression of fetal hemoglobin
Source: eLife. 2022 Feb 11;11:e65421. doi: 10.7554/eLife.65421 (PMC8865852; doi:10.7554/eLife.65421)
Supplement: Supplementary file 1. [file elife-65421-supp1.docx]

**Supplementary file 1:** **The gRNAs used in this study to screen the *HBG* promoter region and their respective primer for sequencing**

| **Sl.no** | **Names** | **gRNA Info 5'-3'** | **Editing window for CBE** | **Editing window for ABE** | **Primers used for Sanger sequencing** | **Primers used for NGS** | **Primers used for HBG1/HBG2 separation** |
| --- | --- | --- | --- | --- | --- | --- | --- |
| 1 | gRNA 1 | ggctagggatgaagaataaa | yes | yes | HBF 1 F/HBF1R | NGS 2 F/R | Not applicable |
| 2 | gRNA 2 | cttgaccaatagccttgaca | yes | yes | HBF 1 F/HBF1R | NGS 2 F/R | NGS4 F/NGS 2 R |
| 3 | gRNA 3 | atAtttgcattgagatagtg | yes | yes | HBF 1 F/HBF1R | NGS 2 F/R | NGS4 F/NGS 2 R |
| 4 | gRNA 4 | gtggggaaggggcccccaag | nil | yes | HBF 1 F/HBF1R | NGS 2 F/R | NGS4 F/NGS 2 R |
| 5 | gRNA 5 | tggtcaagtttgccttgtca | yes | yes | HBF 1 F/HBF1R | NGS 2 F/R | Not applicable |
| 6 | gRNA 6 | gtttgccttgtcaaggctat | yes | nil | HBF 1 F/HBF1R | NGS 2 F/R | Not applicable |
| 7 | gRNA 7 | cttgtcaaggctattggtca | yes | yes | HBF 1 F/HBF1R | NGS 2 F/R | Not applicable |
| 8 | gRNA 8 | caaggctattggtcaaggca | yes | yes | HBF 1 F/HBF1R | NGS 2 F/R | Not applicable |
| 9 | gRNA 9 | gctattggtcaaggcaaggc | nil | yes | HBF 1 F/HBF1R | NGS 2 F/R | Not applicable |
| 10 | gRNA 10 | aggcaaggctggccaaccca | yes | yes | HBF 1 F/HBF1R | NGS 2 F/R | NGS4 F/NGS 2 R |
| 11 | gRNA 11 | ggcaaggctggccaacccat | yes | yes | HBF 1 F/HBF1R | NGS 2 F/R | NGS4 F/NGS 2 R |
| 12 | gRNA 12 | aaggctggccaacccatggg | yes | nil | HBF 1 F/HBF1R | NGS 2 F/R | Not applicable |
| 13 | gRNA 13 | cccatgggtggagtttagcc | yes | yes | HBF 1 F/HBF1R | NGS 2 F/R | NGS4 F/NGS 2 R |
| 14 | gRNA 14 | ccatgggtggagtttagcca | nil | yes | HBF 1 F/HBF1R | NGS 2 F/R | NGS4 F/NGS 2 R |
| 15 | gRNA 15 | gctaaactccacccatgggt | yes | yes | HBF 1 F/HBF1R | NGS 2 F/R | NGS4 F/NGS 2 R |
| 16 | gRNA 16 | ccctggctaaactccaccca | yes | yes | HBF 1 F/HBF1R | NGS 2 F/R | NGS4 F/NGS 2 R |
| 17 | gRNA 17 | tatctgtctgaaacggtccc | yes | nil | HBF 1 F/HBF1R | NGS 2 F/R | Not applicable |
| 18 | gRNA 18 | tatttgcattgagatagtgt | yes | yes | HBF 1 F/HBF1R | NGS 2 F/R | Not applicable |
| 19 | gRNA 19 | atgcaaatatctgtctgaaa | yes | Yes | HBF 1 F/HBF1R | NGS 2 F/R | NGS4 F/NGS 2 R |
| 20 | gRNA 20 | ggaatgactgaatcggaaca | yes | yes | HBF 2 F/HBF1R | NGS 3 F/R | NGS4 F/NGS 2 R |
| 21 | gRNA 21 | actgaatcggaacaaggcaa | yes | yes | HBF 2 F/HBF1R | NGS 3 F/R | NGS4 F/NGS 2 R |
| 22 | gRNA 22 | aaaaactggaatgactgaat | yes | yes | HBF 2 F/HBF1R | NGS 3 F/R | Not applicable |
| 23 | gRNA 24 | gcattgagatagtgtgggga | nil | yes | HBF 2 F/HBF1R | NGS 2 F/R | Not applicable |
| 24 | gRNA 25 | attgagatagtgtggggaag | nil | yes | HBF 2 F/HBF1R | NGS 2 F/R | Not applicable |
| 25 | gRNA 29 | agaataaattagagaaaaac | nil | yes | HBF 2 F/HBF1R | NGS 3 F/R | Not applicable |
| 26 | gRNA 30 | ggagaaggaaactagctaaa | nil | yes | HBF 2 F/HBF1R | NGS 3 F/R | NGS4 F/NGS 2 R |
| 27 | gRNA 32 | cagttccacacactcgcttc | yes | yes | HBF 1 F/HBF1R | NGS 2 F/R | NGS4 F/NGS 2 R |
| 28 | gRNA 33 | cttcatccctagccagccgc | yes | yes | HBF 1 F/HBF1R | NGS 2 F/R | Not applicable |
| 29 | gRNA 34 | cctagccagccgccggcccc | yes | yes | HBF 1 F/HBF1R | NGS 2 F/R | NGS4 F/NGS 2 R |
| 30 | gRNA 35 | ccgccggcccctggcctcac | yes | nil | HBF 1 F/HBF1R | NGS 2 F/R | Not applicable |
| 31 | gRNA 36 | actggatactctaagactat | yes | yes | HBF 1 F/HBF1R | NGS 2 F/R | NGS4 F/NGS 2 R |
| 32 | gRNA 37 | ccaggggccggcggctggct | yes | yes | HBF 1 F/HBF1R | NGS 2 F/R | Not applicable |
| 33 | gRNA 38 | tgaggccaggggccggcggc | yes | yes | HBF 1 F/HBF1R | NGS 2 F/R | Not applicable |
| 34 | gRNA 39 | ttagagtatccagtgaggcc | nil | yes | HBF 1 F/HBF1R | NGS 2 F/R | Not applicable |
| 35 | gRNA 40 | tagtcttagagtatccagtg | yes | yes | HBF 1 F/HBF1R | NGS 2 F/R | NGS4 F/NGS 2 R |
| 36 | gRNA 41 | tagagtatccagtgaggcca | yes | yes | HBF 1 F/HBF1R | NGS 2 F/R | Not applicable |
| 37 | gRNA 42 | agagtatccagtgaggccag | yes | yes | HBF 1 F/HBF1R | NGS 2 F/R | NGS4 F/NGS 2 R |
| 38 | gRNA 43 | ccagtgaggccaggggccgg | nil | yes | HBF 1 F/HBF1R | NGS 2 F/R | Not applicable |
| 39 | gRNA 44 | caggggccggcggctggcta | yes | nil | HBF 1 F/HBF1R | NGS 2 F/R | NGS4 F/NGS 2 R |
| 40 | gRNA 45 | aagcagcagtatcctcttgg | yes | yes | HBF 2 F/HBF1R | NGS 3 F/R | Not applicable |
| 41 | gRNA 46 | attaagcagcagtatcctct | yes | yes | HBF 2 F/HBF1R | NGS 3 F/R | Not applicable |
| 42 | Control | Plasmid without gRNA | - | - | HBF 1 F, 2 F/HBF1R | NGS 2 and NGS 3 | NGS4 F/NGS 2 R |
