# Supplementary material for: Identification of novel HPFH-like mutations by CRISPR base editing that elevate the expression of fetal hemoglobin
Source: eLife. 2022 Feb 11;11:e65421. doi: 10.7554/eLife.65421 (PMC8865852; doi:10.7554/eLife.65421)
Supplement: Supplementary file 2. [file elife-65421-supp2.docx]

**Supplementary file 2: All the PCR primers, qRT PCR primers and probes used in this study**

| **Sl.no** | | **Primer name** | **Primer sequence 5’- 3’** | **Amplicon size** | **Source** | |
| --- | --- | --- | --- | --- | --- | --- |
| **Primers to confirm the gRNA clones** | | | | | | |
| 1 | | Sequencing F | gagggcctatttcccatgat | 539bp | This study | |
| 2 | | Sequencing R | tggatctctgctgtccctgt |  | This study | |
| **Sanger sequencing primer for *HBG* promoter region** | | | | | | |
| 3 | | HBF 1 F | acaaaagaagtcctggtatc | 490bp | This study | |
| 4 | | HBF 2 F | ttactgcgctgaaactgtgg | 772bp | This study | |
| 5 | | HBF 1 R | cttcccagggtttctcctcc |  | This study | |
| **qRT PCR primers to amplify globin gene expression** | | | | | | |
| 6 | | HBB E2E3 RT F | acctttgccacactgagtgag | 110bp | This study | |
| 7 | | HBB E2E3 RT R | tttgccaaagtgatgggcca |  | This study | |
| 8 | | HBA RT F | cgacaagaccaacgtcaagg | 99bp | This study | |
| 9 | | HBA RT R | gtggggaaggacaggaacat |  | This study | |
| 10 | | HBF E2E3 RT F | cttccttgggagatgccata | 136bp | This study | |
| 11 | | HBF E2E3 RT R | aaaacggtcaccagcacatt |  | This study | |
| 12 | | GAPDH E7E8 RT F | ctgcaccaccaactgcttag | 110bp | This study | |
| 13 | | GAPDH E7E8 RT R | gtcttctgggtggcagtgat |  | This study | |
| **NGS primers for *HBG* promoter region** | | | | | | |
| 14 | | NGS 2 F | gctcttccgatct tgaatcggaacaaggcaaagg | 325bp | This study | |
| 15 | | NGS 2 R | gctcttccgatct gtgaaatgacccatggcgtc |  | This study | |
| 16 | | NGS 3 F | gctcttccgatct cctggacctatgcctaaaaca | 318bp | This study | |
| 17 | | NGS 3 R | gctcttccgatct agtttagccagggaccgttt |  | This study | |
| 18 | | NGS 4 F | gctcttccgatct cggctgacaaaagaagtcct |  | This study | |
| **Primers to differentiate *HBG1* and *HBG2*** | | | | | | |
| 19 | | HBG1 F | ccacagtacctgccaaagaa | 940bp | This study | |
| 20 | | HBG2 F | ccatagtatctggtaaagagca | 940bp | This study | |
| 21 | | HBG1/2 R | ggcgtctggactaggag |  | This study | |
| **Sanger sequencing primer for *AAVS1* locus** | | | | | | |
| 22 | | AAVS1 seqF | gagatggctccaggaaatgg | 420bp | Matthew J.J et al., Sci Rep,2018. | |
| 23 | | AAVS1 seqR | acctctcactcctttcatttgg |  | Matthew J.J et al., Sci Rep,2018. | |
| **ABE 8e lenti plasmid cloning primers** | | | | | | |
| 24 | | ABE 8e F | acacaggtgtcgtgacgcgggatccgccaccatgaaacggacagccgacgg | 4872bp | This study | |
| 25 | | ABE 8e R | agttggtggcgccgctgccgctagcgactttcctcttcttcttgg |  | This study | |
| **qRT PCR primers to validate Vector copy number** | | | | | | |
| 26 | | Cas 9 var RT F | ccgaagaggtcgtgaagaag | 128bp | This study | |
| 27 | | Cas 9 var RT R | gccttatccagttcgctcag |  | This study | |
| 28 | | U6 RT F | agggcctatttcccatgatt | 151bp | This study | |
| 29 | | U6 RT R | aaactgcaaactacccaagaaa |  | This study | |
| 30 | | WREP RT F | caccacctgtcagctccttt | 135bp | This study | |
| 31 | | WREP RT R | acaacaccacggaattgtca |  | This study | |
| 32 | | HBB locus ctrl F | ttggacccagaggttctttg | 123bp | This study | |
| 33 | | HBB locus ctrl F | gagccaggccatcactaaag |  | This study | |
| **qRT PCR primers to validate 4.9kb large deletion** | | | | | | |
| 34 | | Del_qPCR_F | aggggctcaacgaagaaaaagtgt |  | Chang Li et al., Blood, 2018. | |
| 35 | | Del_qPCR_R | cacttcattgtagttaccgtggaaaga |  | Chang Li et al., Blood, 2018. | |
| 36 | | Del_ctrl_qPCR_F | aaatgaatcagcagaggctcac |  | Chang Li et al., Blood, 2018. | |
| 37 | | Del_ctrl_qPCR_R | atgcactaacatccaactatacaaaa |  | Chang Li et al., Blood, 2018. | |
| **Probes used for EMSA** | | | | | | |
| 38 | | HBG2 -110 to -132 Wild type (WT) | ggccagccttgccttgaccaata | Sense | This study | |
| 39 | | HBG2 -110 to -132 Wild type (WT) | tattggtcaaggcaaggctggcc | Antisense | This study | |
| 40 | | HBG2 -110 to -132 -123T>C/-124T>C | ggccagccccgccttgaccaata | Sense | This study | |
| 41 | | HBG2 -110 to -132 -123T>C/-124T>C | tattggtcaaggcggggctggcc | Antisense | This study | |
| 42 | | Hbbt1 CACCC (KLF1 +ve control) | tagagccacaccctggtaag | Sense | Merlin C et al., Mol Cell Biol, 1996. | |
| 43 | | *Hbbt1 CACCC (KLF1 +ve control)* | cttaccagggtgtggctcta | Antisense | Merlin C et al., Mol Cell Biol, 1996. | |
| **qRT PCR primers used for ChIP analysis** | | | | | | |
| 44 | | *γ-globin* promoter -73 to -179 F | caaatatctgtctgaaacggtccc | 106 bp | Gabriella E. M et al., Blood, 2019. | |
| 45 | | *γ-globin* promoter -73 to -179 R | actctaagactattggtcaagtttgc |  | Gabriella E. M et al., Blood, 2019. | |
| 46 | | *γ-globin* promoter -199 to -106 F | tcaatgcaaatatctgtctgaaacg | 93bp | Gabriella E. M et al., Blood, 2019. | |
| 47 | | *γ-globin* promoter -199 to -106 R | caaggctattggtcaaggcaa |  | Gabriella E. M et al., Blood, 2019. | |
| 48 | | *SP1* promoter F | acctctccgcccactagga |  | Beeke Wienert et al., Blood, 2017. | |
| 49 | | *SP1* promoter R | caacggccaaccagaatcc |  | Beeke Wienert et al., Blood, 2017. | |
| 50 | | *VegfA* F | ggtttgtatcctgcccttc |  | Beeke Wienert et al., Blood, 2017. | |
| 51 | | *VegfA* R | actgggtcttgctgttttcc |  | Beeke Wienert et al., Blood, 2017. | |
| **Control gRNA for CD34+ cells editing** | | | | | | |
| 52 | | AAVS1 | ggggccactagggacaggat |  | Matthew J.J et al., Sci Rep,2018. | |
| **Primers to amplify DNA off-target** | | | | | | |
| 53 | G11 OT1 F | | tacacgacgctcttccgatct ggcagaggggacacatcagt | 192bp | | This study |
| 54 | G11 OT1 R | | agacgtgtgctcttccgatct tgcgctatgatgttgggtatgtcc |  | | This study |
| 55 | G11 OT2 F | | tacacgacgctcttccgatct acttaatgctcacagattggttcaatcaggc | 190bp | | This study |
| 56 | G11 OT2 R | | agacgtgtgctcttccgatct agccaatcaggttcaagatggcag |  | | This study |
| 57 | G11 OT3 F | | tacacgacgctcttccgatct gtctactggaaagcccatttgcatagtaag | 200bp | | This study |
| 58 | G11 OT3 R | | agacgtgtgctcttccgatct gtgagagagagaggacttctgag |  | | This study |
| 59 | G11 OT4 F | | tacacgacgctcttccgatct tcctcagttccaagccttggg | 194bp | | This study |
| 60 | G11 OT4 R | | agacgtgtgctcttccgatct gtcatccccaatccacaaactcac |  | | This study |
| 61 | G11 OT5 F | | tacacgacgctcttccgatct cacacgtgtcttatctgtcacctc | 193bp | | This study |
| 62 | G11 OT5 R | | agacgtgtgctcttccgatct ctgaccactcttgcaactccatg |  | | This study |
| 63 | G11 OT6 F | | tacacgacgctcttccgatct atcagggaaaaacctgccatgtgc | 191bp | | This study |
| 64 | G11 OT6 R | | agacgtgtgctcttccgatct gcaattcctctgttcaagacaggatgtag |  | | This study |
| 65 | G11 OT7 F | | tacacgacgctcttccgatct gggcaaggggaaacttcatttgca | 192bp | | This study |
| 66 | G11 OT7 R | | agacgtgtgctcttccgatct gagaggcgacttctgagagg |  | | This study |
| 67 | G11 OT8 F | | tacacgacgctcttccgatct ttacaggcatgaaccaccacatcc | 194bp | | This study |
| 68 | G11 OT8 R | | agacgtgtgctcttccgatct gtcagagaactgagcctaatggag |  | | This study |
| 69 | G11 OT9 F | | tacacgacgctcttccgatct cacttgagatcaggatttggagaccag | 192bp | | This study |
| 70 | G11 OT9 R | | agacgtgtgctcttccgatct gcgcaatcttgggtcactgca |  | | This study |
| 71 | G11 OT10 F | | tacacgacgctcttccgatct ggcccacagattggttagatcag | 194bp | | This study |
| 72 | G11 OT10 R | | agacgtgtgctcttccgatct ctacttgggactaggcatgttcag |  | | This study |
| 73 | G11 OT11 F | | tacacgacgctcttccgatct gggggctactctaataaaaagactttaggaac | 200bp | | This study |
| 74 | G11 OT11 R | | agacgtgtgctcttccgatct gttccttatcagcttatgtagattttgggctgag |  | | This study |
