# Supplementary material for: Identification of novel HPFH-like mutations by CRISPR base editing that elevate the expression of fetal hemoglobin
Source: eLife. 2022 Feb 11;11:e65421. doi: 10.7554/eLife.65421 (PMC8865852; doi:10.7554/eLife.65421)
Supplement: Supplementary file 3. [file elife-65421-supp3.docx]

**Supplementary file 3: The targets analyzed for DNA off-target**

| **Sl.no** | **Name** | **DNA offtarget target seq with PAM** | **Chromosome** | **Position** | **Mismatches** |
| --- | --- | --- | --- | --- | --- |
| 1 | OT1 | ttgggttgcccagacttgccagg | chr1 | 25039865 | 3 |
| 2 | OT2 | gtggggtggccagcctttcctgg | chr1 | 235427633 | 3 |
| 3 | OT3 | gtggggtggccagccttccctgg | chr2 | 64808323 | 3 |
| 4 | OT4 | atggggtgtgcagccttgcctgg | chr12 | 113177162 | 3 |
| 5 | OT5 | aggggttggcccgccttcccagg | chr16 | 674938 | 3 |
| 6 | OT6 | ttggcttggccagacttgcccgg | chr9 | 110575343 | 3 |
| 7 | OT7 | gtggggtggccagccttcccagg | chr9 | 118816545 | 3 |
| 8 | OT8 | atgggttgtccagcgttgcttgg | chrX | 150946920 | 3 |
| 9 | OT9 | atgggttggccaggctggtctgg | chr6 | 7187976 | 3 |
| 10 | OT10 | gtggggtggccagccttccctgg | chr10 | 119547882 | 3 |
| 11 | OT11 | atgggttgaccaacctggccagg | chr13 | 86143790 | 3 |
